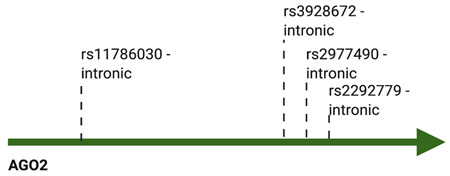# Argonaute Proteins Take Center Stage in Cancers

**DOI:** 10.3390/cancers13040788

**Published:** 2021-02-13

**Authors:** Iwona Nowak, Aishe A. Sarshad

**Affiliations:** 1 Department of Medical Biochemistry and Cell Biology, Institute of Biomedicine, The Sahlgrenska Academy, University of Gothenburg, 405 30 Gothenburg, Sweden; Iwona.nowak@gu.se; 2 Wallenberg Centre for Molecular and Translational Medicine, University of Gothenburg, 405 30 Gothenburg, Sweden

**Keywords:** Argonaute, miRNA, cancer, tumorigenesis, biomarker, therapeutics

## Abstract

**Simple Summary:**

The dysregulation of RNA interference (RNAi) has often been observed in cancers, where the main focus of research has been on the small RNA molecules directing RNAi. In this review, we focus on the activity of Argonaute proteins, central components of RNAi, in tumorigenesis, and also highlight their potential applications in grading tumors and anti-cancer therapies.

**Abstract:**

Argonaute proteins (AGOs) play crucial roles in RNA-induced silencing complex (RISC) formation and activity. AGOs loaded with small RNA molecules (miRNA or siRNA) either catalyze endoribonucleolytic cleavage of target RNAs or recruit factors responsible for translational silencing and target destabilization. miRNAs are well characterized and broadly studied in tumorigenesis; nevertheless, the functions of the AGOs in cancers have lagged behind. Here, we discuss the current state of knowledge on the role of AGOs in tumorigenesis, highlighting canonical and non-canonical functions of AGOs in cancer cells, as well as the biomarker potential of AGO expression in different of tumor types. Furthermore, we point to the possible application of the AGOs in development of novel therapeutic approaches.

## 1. Introduction

RNA interference (RNAi) plays a crucial role in post-transcriptional regulation of gene expression. In mammalian cells, RNAi is mediated by three classes of small RNA (smRNA), approximately 20–25 nt long, endogenous piwiRNA (piRNA), microRNAs (miRNAs) or artificial small interfering RNAs (siRNAs). These smRNAs are loaded to a member of the Argonaute (AGO) protein family, which comprises an AGO subclass (loaded with si/miRNA) and PIWI subclass (loaded with piRNA) [1]. Together, they form the core of the RNA-induced silencing complex (RISC) [2]. Since the discovery of RNAi, AGO involvement in RISC assembly and small RNA (smRNA) maturation has been extensively studied [3]. However, the complex network of AGO–miRNA interactions, promoting the maturation and function of specific miRNAs, is still largely unexplored. As a core component of the RISC ribonucleoprotein complex, AGO proteins hold a great importance in fine tuning cellular protein and RNA profiles in order to ensure normal development and homeostasis [1]. It is now estimated that approximately 60% of protein coding genes in human cells are controlled in an RNAi-dependent manner [4].

**Figure 1:**
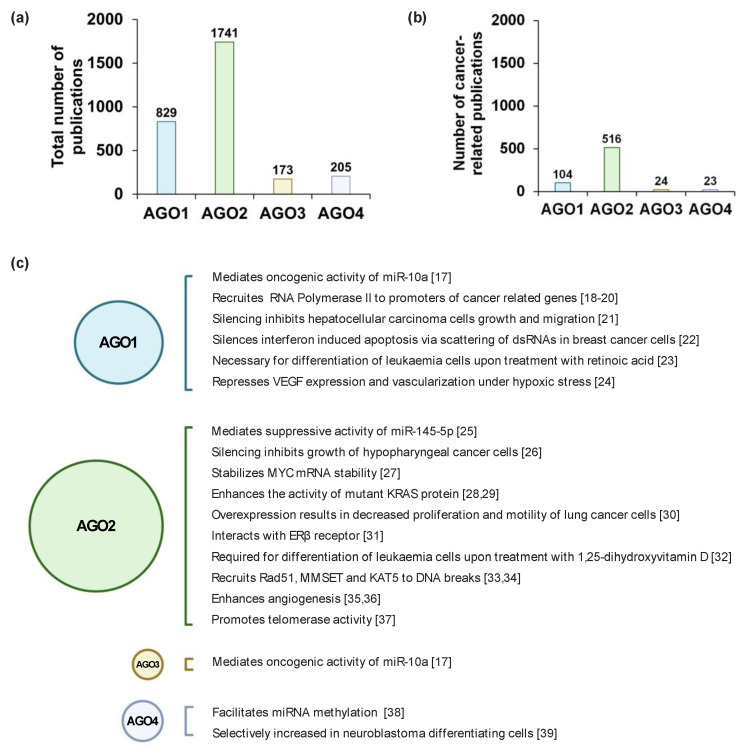
Argonaute (AGO) related research. (**a**) The total number and (**b**) the number of cancer-related studies of AGO1–4 found in PubMed using keywords “AGO1/AGO2/AGO3/AGO4” and “cancer”, until 13 January 13 2021. (**c**) Highlighted studies that show the detailed molecular mechanisms of AGO1–4 in cancer-related processes, based on ref [17,18,19,20,21,22,23,24,25,26,27,28,29,30,31,32,33,34,35,36,37,38,39]. Created with BioRender.com (accessed date: 21 January 2021).

miRNAs are abundantly expressed in all mammalian cell types and at every stage of development. However, the smRNA profiles greatly differ spatiotemporally, which is characteristic for each cellular lineage throughout maturation [5]. In such a manner, AGO1–4 protein expression is tightly controlled during development. Normally, the AGOs are expressed in a tissue-specific manner where, for example, AGO2 is highly expressed in trachea but to a much lesser degree in kidneys [6]. Generally, AGO1 and AGO2 expression is significantly more prominent as compared to the remaining AGO proteins [7,8]; hence, the volume of published data on AGO1 and AGO2 involvement in tumorigenesis is relatively large compared to AGO3 and AGO4 publications (Figure 1a,b). Furthermore, the ratio of different AGO protein levels, with the emphasis on AGO1 and AGO2, are tightly controlled during early stages of mammalian embryonic development [9]. The expression of the AGO proteins changes along the development of organs, exemplified by elevation of AGO1–4 levels in the fetal brain as compared to adult tissue [7].

The dysregulation of RNAi processes has significant negative impacts on cellular homeostasis and has frequently been reported in neoplasia. miRNAs are often dysregulated in cancers [10,11,12]. In fact, it was reported that there is a global downregulation of miRNA expression in cancer tissues [12]. Numerous miRNA molecules have been described as either cancer promoting oncogenes, referred to as oncomiRs, or cancer demoting tumor suppressors [10,11,12,13,14,15]. Moreover, similarly to normal tissues, cancers derived from different origins are characterized by various miRNA dysregulations. Of note, miRNAs exhibiting tumor suppressive activity in one type of cancer may promote development of another [16].

AGO proteins are viewed as the mediators of miRNA function rather than the star players of RNAi, and therefore, in much of the literature, documenting the impact of RNAi dysregulation in tumorigenesis focuses on miRNAs and not their partner proteins. Nevertheless, given the crucial importance of RNAi in the maintenance of cellular homeostasis, AGO proteins are inevitably engaged in cancer development and progression. Hence, unsurprisingly, the 2010s brought a substantial number of reports documenting AGO protein function, dysregulated expression, and mutations in cancer cells and tissues (Figure 1c). Herein, we provide a scrutinized summary of the relationship between AGO1–4 proteins and tumorigenesis.

## 2. The Functional Role of AGO Proteins in RISC

The AGO subclass includes four members, AGO1–4, each exhibiting high affinity to miRNA molecules and able to form functional RISC complexes [40]. The AGO proteins are characterized by four domains: amino-terminal (N), PAZ (PIWI-ARGONAUTE-ZWILLE), MID (middle) and PIWI domains. The PAZ and MID domains are involved in miRNA binding, via anchoring of 5′ and 3′ end of the smRNA molecule, respectively. In mammalian cells, AGO1–4 proteins mediate miRNA-dependent translational repression. Moreover, AGO2, and, to a limited extent, AGO3, possess endoribonuclease activity, which can catalyze smRNA-directed ribonucleolytic cleavage of target mRNAs [41,42,43].

Mature miRNAs and siRNAs are single stranded; however, AGO proteins recognize and bind their double-stranded precursors, containing functional guide and passenger strands (the latter is subsequently discarded). Therefore, AGO proteins are crucial not only for the functionality but also maturation of smRNAs. Formation of miRNA:AGO complexes are done in two steps—loading, which is facilitated by TRBP and DICER proteins, and unwinding of the double-stranded RNA [1]. The mechanisms of the unwinding process in mammalian cells remains elusive, because the partner proteins involved in passenger strand removal and degradation are yet to be identified [2].

After disposal of the passenger strand, mature miRNA or siRNA molecules guide the AGO protein to complementary sequences in the transcriptome. For siRNAs, the complementarity with the target sequence is complete; in the case of miRNA-mediated gene silencing, only partial base pairing is necessary. The mechanism of smRNA-mediated negative regulation of gene expression is dependent on the specific AGO protein enrolled in the RISC formation. Out of the four AGO proteins, only AGO2 is equipped with a fully functional endoribonucleolytic domain and is able to directly cleave target sequences with extensive complementarity to the bound siRNA. The function of RISC, containing the remaining AGO proteins, is exerted through the interaction with partner proteins, facilitating the destabilization of the target transcript or its translational inhibition. Further detailed mechanism of canonical RISC function will not be discussed here, because it has been thoroughly reviewed elsewhere [1,44,45,46].

## 3. The Involvement of AGO Proteins in Tumor Associated Processes

**Figure 2:**
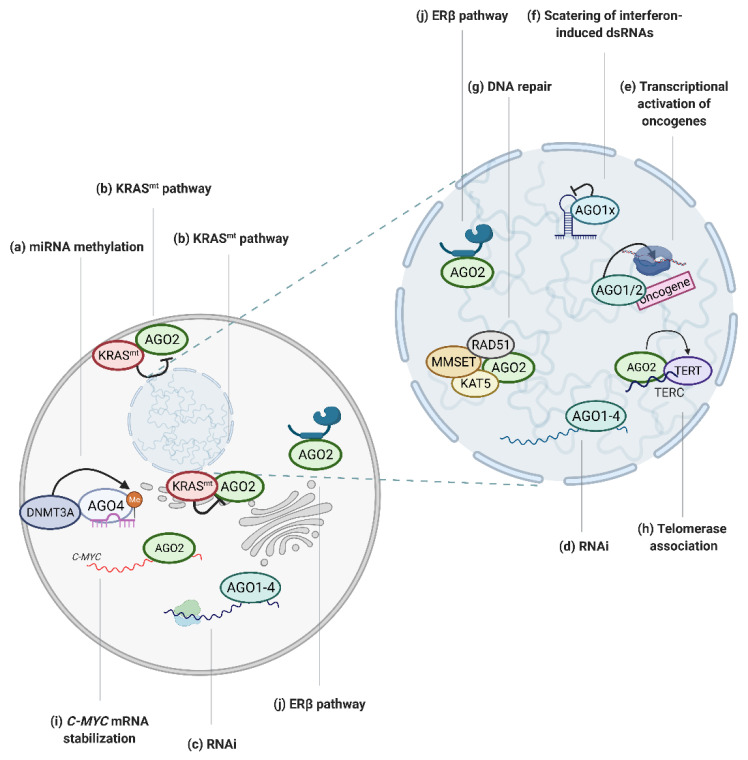
Graphical depiction of AGO-regulated tumor associated processes. (**a**) AGO4 enhances miRNA methylation, which inhibits miRNA activity [38]; (**b**) mutated KRAS protein negatively regulates AGO2 activity [28,29]; (**c**) AGO proteins mediate RNAi in cytoplasm [1] and (**d**) nucleus [47] in cancer cells; (**e**) AGO1 and AGO2 bind to promoters of oncogenes to activate transcription [18,19,20]; (**f**) AGO1 translational read-through variant, AGO1x, inhibits interferon-induced apoptosis via the depletion of nuclear dsRNA [22]; (**g**) AGO2 recruits RAD51, MMSET and KAT5 proteins to dsDNA breaks to facilitate DNA repair [33,34]; (**h**) AGO2 promotes telomerase activity [37]; (**i**) AGO2 tethers *MYC* mRNA and increases its stability [27]; (**j**) AGO2 activity is regulated via ERβ [31]. Created with BioRender.com (accessed date: 21 January 2021).

The literature regarding the dysregulation of RNAi factors in pathological states focuses at large on the smRNA molecules directing RNAi. However, it is important to consider that smRNAs alone cannot catalyze any chemical reactions. Therefore, the activity and function of smRNAs must be considered with regards to their partner proteins, AGO1–4. In fact, numerous reports have shed new light on the involvement of canonical and non-canonical AGO functions in tumorigenesis (Figure 2).

### 3.1. Regulation of AGO:miRNAs by Post-Transcriptional Modifications

The activity of AGO proteins is, to a degree, regulated via post-transcriptional modifications (PTMs) including prolyl-4-hydroxylation, phosphorylation, ubiquitination, poly-ADP-ribosylation (PARylation) and SUMOylation [40]. PTMs of AGOs are fine-tuned by numerous proteins, which are also dysregulated in pathological states, such as cancer. For instance, AGO2 is phosphorylated by a protein product of the well-characterized oncogene, *EGFR*, at Y393, which weakens AGO2: DICER interaction, resulting in the inhibition of miRNA maturation [48]. This phenomenon was found to be enhanced under hypoxia, a stress state often observed in solid tumors [48] and characterized by low oxygen levels. Moreover, phosphorylation is catalyzed by CSNK1A1 kinase, an implicated oncoprotein in leukaemia, at residues S824–S834, which negatively affects the interaction of RISC with target transcripts [49,50,51,52]. AGO2 is further regulated via acetylation at K355, K493, and K720 exerted by P300/CBP complex [53]. Two of these PTMs (acetylation at K493 and K270, but not K355) facilitate enhanced maturation of miR-19b, an oncogenic miRNA [53]. The P300/CBP/AGO2/miR-19b axis promotes growth and proliferation of A549, a lung cancer cell line, and in vivo lung cancer xenograft mouse model [53]. Moreover, mitogenic Akt3 kinase phosphorylates AGO2 protein at S387, resulting in the switch of AGO2-RISC activity from mRNA cleavage to translational repression and increased localization of the complexes to P-bodies, cytoplasmic foci of mRNA turnover [54,55]. Furthermore, this PTM was found to be dependent on the oncogenic KRAS/MEK/ERK signaling pathway, which negatively affects the sorting of AGO2 protein to exosomes [56].

The activity of RISC in mammalian cells may also be regulated by modifications of the miRNAs, such as cytosine methylation, which inhibits miRNA:target mRNA binding [57]. AGO4 has been shown to facilitate the recruitment of DNMT3A methyltransferase to the associated miRNA, thus enhancing miRNA methylation (Figure 2a) [38]. Interestingly, high methylation of the tumor suppressor miR-181a-5p is correlated with poor outcomes of glioblastoma multiforme [38]. Hence, AGO4 may contribute to neoplasia formation via promotion of miRNA cytosine methylation, and thus inhibition of tumor suppressor miRNAs.

### 3.2. Regulation of AGOs by Protein/RNA Co-Factors

The RISC machinery in cancer can be regulated by miRNAs. The production of AGO2 was found to be attenuated by miR-99a in hepatocellular carcinoma cells [58]. Moreover, there was a notable reduction in hepatocellular carcinoma tumorigenicity upon miR-99a overexpression or AGO2 silencing, which points to an oncogenic potential of AGO2 [58]. Another layer of regulation of RISC in physiological and pathological states is executed by the ratios of AGO1–4 protein content in the cell, because the function of specific miRNA molecules may depend on the type of AGO protein to which it is bound [25,59]. The suppressive activity of miR-145-5p is, to exemplify, manifested only in RISC complexes with AGO2 and not the remaining AGO proteins [25]. On the other hand, oncogenic miR-10a is able to negatively regulate target gene expression only in complex with AGO1 and AGO3 [59]. In this manner, the impact of dysregulation of specific AGO proteins on tumorigenesis may vary.

Apart from being an RNA binding protein, the function of AGO proteins is dependent on their interactions with protein co-factors, for some of which RNAi is not the main function. For instance, in multiple myeloma cells, AGO2 is destabilized upon binding with cereblon, a therapeutic target of immunomodulatory drugs [60]. The interaction markedly contributed to cytotoxicity of the drugs [60]. Furthermore, AGO2 was found to be an important binding partner of the protein product of *KRAS*, a well-characterized oncogene (Figure 2b) [28,29]. It was shown that high levels of AGO2 protein enhanced neoplastic transformation driven by KRAS mutants, whereas knockout of AGO2 resulted in the growth arrest of KRAS-dependent cancer cells [28]. Moreover, AGO2-mutated KRAS interaction holds crucial importance in pancreatic ductal adenocarcinoma progression, where AGO2 expression is required for overcoming oncogene-induced senescence and cancer development [29].

### 3.3. AGOs in the Nucleus Affect Cancer Progression

In the early days of RNAi research, the general consensus was that RISC-mediated gene silencing is limited to cytoplasmic transcripts, particularly mature mRNAs (Figure 2c) [61,62]. However, a growing body of data documents the presence of functional RISC components in cell nuclei [17,47,63,64]. Even though miRNA loading factors are absent from the nucleus, RNAi factors associate into active complexes (Figure 2d) [47,63], Furthermore, the assessment of miRNA binding sites in stem cell nuclei revealed that approximately 50% of the miRNAs are bound to intronic regions of pre-mRNAs [47]. The significance of nuclear RNAi for tumorigenesis, however, remains largely unexplored.

Beyond RNAi, the suggested involvement of the AGOs in nuclear processes spans from chromatin remodeling to transcriptional regulation, splicing, DNA repair and regulation of telomerase activity [40]. Generally, in mammalian cell nuclei, the literature points to AGO-mediated negative transcriptional regulation via the recruitment of either protein or DNA methyltransferases to promoter regions of the target genes by the AGO proteins. The interaction is followed by heterochromatin formation and inhibition of the gene transcriptional activity via similar mechanisms as those described in yeast and drosophila [65,66,67,68,69,70,71]. Interestingly, in some cases, smRNA-directed AGO proteins can bind to promoter regions to facilitate the recruitment of RNA Polymerase II and gene transcription (Figure 2e) [72,73,74,75]. For instance, the involvement of AGO2 in transcriptional activation was described in breast cancer cells, in which AGO2 was found to promote cell growth by inducing mRNA synthesis of the progesterone receptor (PR) by binding to the promoter region of the *PR* gene [76].

In human cancer cells, the involvement of AGO1 in chromatin remodeling has been reported [19,20]. The protein was found to enhance the recruitment of RNA Polymerase II to promoters of genes involved in cell growth and survival in prostate cancer cells [18]. Moreover, AGO1-mediated transcriptional activation of cancer related genes, in a manner dependent on AT repeats at the promoter region, was implicated in neoplasia of various origin [19]. Moreover, one report points to the tumor-promoting activity of AGO1x, a translational readthrough variant of AGO1, dependent on nuclear scattering of dsRNAs and silencing of interferon-induced apoptosis in breast cancer cells (Figure 2f) [22].

### 3.4. Regulation of DNA Integrity

The correctness of the cellular genome is ensured via the DNA damage response (DDR) pathway [77]. Depending on the severeness of the genetic aberrations, either repair mechanisms are triggered or the cell is directed towards senescence or apoptosis [77]. The rapid growth and divisions of cancer cells, characterized by numerous genetic aberrations, is reliant on the dysregulation of the DDR pathway [77]. DDR is partially regulated via smRNAs, which are induced by double-stranded DNA breaks [33]. The smRNAs are recognized by AGO2 protein, which in turn recruits Rad51 and enhances DNA repair via homologous recombination (Figure 2g) [33]. Furthermore, it was suggested that AGO2, loaded with smRNAs, facilitates the enrolment of methyltransferase MMSET (WHSC1) and the acetyltransferase Tip60 (KAT5) to sites of double-stranded breaks on the DNA, leading to enhanced Histone H4 di- and tri-methylation at lysine 20 and H4 acetylation at lysine 16 (Figure 2g) [34]. Consequently, these histone modifications lead to an open chromatin configuration, which in turn facilitate the recruitment of the DNA repair machinery to double-stranded DNA breaks [34]. Moreover, the importance of AGO2 in the DDR pathway is manifested by the impairment of the DNA repair potential of AGO2-deficient osteosarcoma cells [78].

The involvement of AGO2 in the regulation of the DDR pathway is not limited to facilitating or triggering repair of double-stranded DNA breaks [77]. Upon recognition of DNA damage, cell fate is decided between DNA repair and death or senescence [77]. The decision is surveilled by P53, a well-characterized tumor suppressor protein [77]. Upon DNA damage, P53 interacts with AGO2 to fine-tune the subset of AGO2 associated miRNAs. One of the notable P53 mediated alteration in the AGO2:miRNA interactome is the enhancement of loading of the suppressive let-7 miRNA family into functional RISC complexes [79].

The integrity of chromosome ends is ensured by telomers, which are nucleoprotein structures localized at chromosome ends containing repetitive nucleotide sequences [80]. Telomeres can be elongated by the telomerase enzyme, which is physiologically only active in human gametes and stem cells [80]. However, in advanced cancers telomerase is reactivated, which results in telomere elongation and strengthens the proliferative potential of cancer cells [81]. Studies in HeLa cells, a cervical cancer cell line, uncovered the interaction between AGO2 and telomerase reverse transcriptase (TERT), as well as the telomerase RNA component (TERC), which promotes TERT and TERC association [37]. TERT and TERC constitute the core of the telomerase enzyme; hence, AGO2-dependentent promotion of binding between the two components pointed to a new role of AGO2 in regulating telomerase activity (Figure 2h). Indeed, AGO2 silencing caused significant shortening of the telomeres in HeLa cells, which further implicated the importance of the protein in telomere length tuning [37].

### 3.5. Cellular Differentiation

Apart from rapid proliferation and cell growth, tumor tissues are characterized by the maintenance of the undifferentiated status of cancer cells. A crucial role of AGO proteins in embryonic development and neoplastic transformation of leukaemic myeloid progenitors has been implicated by numerous research groups [9,23,32,47,82,83]. It was shown that increased expression of AGO2 protein is necessary for monocyte differentiation of leukemic myeloid progenitors, whereas granulocyte differentiation requires maintenance of high AGO1 levels [23,32]. Furthermore, the presence of both AGO1 and AGO2 proteins was essential for the successful induction of differentiation of leukaemia cells upon treatment with retinoic acid or 1,25-dihydroxyvitamin D3, respectively [23,32]. Therefore, high expression of AGO proteins in tumor tissues may be favorable for therapeutic-induced differentiation of cancer cells.

The involvement of AGO proteins in cellular differentiation has also been assessed in neuroblastoma. Potenza et al. reported a selective increase in AGO4 expression in differentiating neuroblastoma cells, whereas the levels of other AGO proteins decreased [39]. Such patterns of AGO protein expression implies crucial importance of AGO4-mediated regulation of gene expression in the differentiation of neuroblastoma cells [39].

### 3.6. Angiogenesis

Inside solid tumors, cancer cells are often under hypoxic stress, which can contribute to tumorigenesis by simulating angiogenesis [84]. The inhibitory effect of AGO2 downregulation on umbilical vein endothelial cell growth and tube formation has been previously reported [85,86]. In fact, the crucial role of AGO2 in stimulating tumor-mediated angiogenesis was demonstrated in hepatocellular carcinoma [35]. The expression of AGO2 in six hepatocellular carcinoma cell lines was correlated with the expression and release of VEGF, a key factor promoting vascularization [35]. Moreover, silencing of AGO2 in these cell lines led to a decrease in VEGF levels [35]. The pro-angiogenesis activity of AGO2 was also described in multiple myeloma, in which AGO2 promoted the secretion of miRNAs, stimulating the formation of new blood vessels [36].

Upon hypoxia, AGO2 function is fine-tuned by post-transcriptional modifications (PTM). In low oxygen conditions, AGO2 is hydroxylated, which stabilizes the protein as well as promotes intracellular activity and release of miR-210 [87,88]. miR-210 exerts pro-angiogenic functions; therefore, AGO2 hydroxylation may contribute to the vascularization of solid tumors [89]. Hydroxylated AGO2 is also recognized by HSP90, which triggers translocation of RISC complexes into stress granules [90]. Another AGO2 hypoxia-mediated PTM is phosphorylation at Y393, which is catalyzed by EGFR. This modification reduces the maturation of miRNA precursors, apart from a specific set of miRNAs involved in the promotion of cell survival and invasiveness, including miR-21 and miR-192 [48].

Despite numerous reports documenting pro-angiogenic activity of AGO2, AGO1 was found to repress VEGF expression and vascularization under hypoxic stress [24]. Moreover, some miRNAs, including miR-103/107 and let-7, are upregulated upon hypoxia and repress AGO1 translation to promote angiogenesis in HUVEC cells [24].

### 3.7. Motility and Metastasis

An important aspect of cancer pathogenesis is the ability of cancer cells to migrate from the place of origin and form metastatic tumors. The mechanisms underlying cancer metastasis have been extensively studied, allowing for identification and characterization of epithelial–mesenchymal transition (EMT), a process enhancing motility and metastatic potential of tumor cells [91]. In hepatocellular carcinomas, a correlation between activation of the EMT process and AGO1 has been identified. It was shown that the depletion of AGO1 in HCCLM3 cell lines resulted in a significant inhibition of migration and downregulation of proteins involved in EMT, which points to the metastasis-promoting activity of AGO1 [21].

AGO2 has also been linked to enhanced metastasis activity in hepatocellular carcinoma. The pro-metastatic activity of AGO2 is not connected with its canonical function, but rather it depends on AGO2-mediated transcriptional activation [92]. Mechanistically, it was shown that synthesis of the focal adhesion kinase (FAK) mRNA, derived from the *FAK* gene promoter, one of the key EMT promoters, is triggered by the binding of AGO2 to the promoter region [92]. Furthermore, reports also point to the potential of metastasis facilitation by AGO2 canonical function. The protein interacts with newly-identified pro-metastatic protein LASP1 in breast cancer cells, in a manner which is dependent on LASP1 phosphorylation by C-X-C chemokine receptor type 4 [93]. This leads to modified activity of the AGO2-associated miRNAs. Most importantly, the interaction between LASP1 and AGO2 causes the inhibition of anti-metastatic let-7 activity [93].

### 3.8. Tumor-Promoting and Anti-Cancer Functions of the AGOs

The oncogenic function of AGO2 was documented in hypopharyngeal cancer where knock down of *AGO2* led to the inhibition of cell growth and tumor formation in mice, as well as activation of the mitogenic FAK/PI3K/AKT pathway [26]. Additionally, AGO2 was found to tether *MYC* mRNA, increasing its stability in hepatocellular cancer cells, and therefore promote cell survival and proliferation (Figure 2i) [27]. Moreover, AGO1 was shown to exhibit tumor-promoting activity in hepatocellular carcinoma cells, which underwent potent proliferation arrest upon AGO1 silencing [21]

Despite growing evidence of oncogenic potential of AGO proteins discussed above, a portion of the published data points to the tumor suppressor activity of AGO2. In fact, overexpression of AGO2 resulted in decreased proliferation and motility of H1299 lung cancer cells [30]. Moreover, AGO2 protein seems to be involved in the negative regulation of FGF2, which is elevated in numerous cancers and contributes to rapid proliferation of cancer cells [94]. Additionally, AGO2 has been found to interact with the ERβ receptor, a protein exerting tumor suppressive functions, which regulates both canonical and non-canonical AGO2 activities (Figure 2j) [31].

## 4. AGO Proteins as Potential Biomarkers

### 4.1. The Prognostic Value of AGO Protein Expression in Cancer

**Figure 3:**
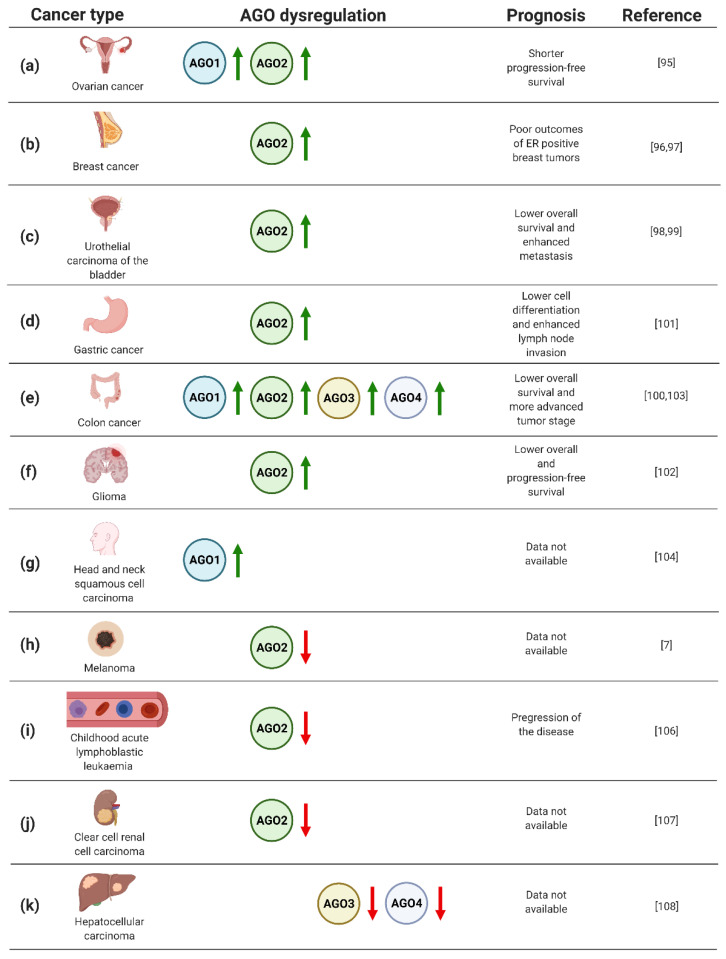
(**a**–**k**) Dysregulation of AGO1–4 in cancers, based on refs [7,95,96,97,98,99,100,101,102,103,104,105,106,107,108]. Created with BioRender.com (accessed date: 21 January 2021).

The dysregulated expression of the genes encoding AGO proteins, with emphasis on AGO2 and, to a lesser extent AGO1, was demonstrated in neoplastic tissues of numerous cancer types (Figure 3). Hence, the biomarker potential of AGO1 and AGO2 in cancers of different origin has been extensively explored, pointing to a prognostic value of AGO proteins in solid tumors as well as leukaemia.

In particular, the analysis of AGO protein expression in 103 ovarian carcinoma specimens uncovered elevated levels of AGO1 and AGO2 in metastatic tumors (Figure 3a) [95]. In addition, high levels of AGO2 mRNA were correlated with shorter progression-free survival (Figure 3a) [95]. Furthermore, a significant number of published data documents the potential of AGO2 as a biomarker in breast cancer [96,97]. Analysis of gene expression data derived from The Cancer Genome Atlas and 291 breast cancer specimens pointed to a correlation of high expression of AGO2 with unfavorable, hormone receptor-positive, subtypes of disease, and poor clinical outcome (Figure 3b) [96,97]. Moreover, the assessment of AGO2 expression profiles resulted in an improvement of prediction of the breast cancer subtype and estrogen receptor or progesterone receptor status by 15–20% [97].

Furthermore, AGO2 upregulation was implicated as an adverse prognostic biomarker for urothelial carcinoma of the bladder [98,99]. Based on the analysis of 106 cases, high expression of AGO2 correlated both with metastasis and lower overall survival of the patients (Figure 3c) [98]. Looking in detail, the assessment of AGO2 expression levels in bladder tissues derived from urothelial carcinoma was sufficient to discriminate between muscle invasive and non-muscle invasive tumors [99]. Moreover, the assessment of AGO2 expression may serve as a prognostic factor for grading patients suffering from colorectal cancer [100]. Evaluation of AGO2 in 76 colorectal tumor samples revealed a significant correlation between an increase in gene expression and progression of disease from II to III stage tumors (Figure 3e) [100]. Similarly, the association of high AGO2 expression and adverse tumor characteristics were also found for gastric cancer [101]. Evaluating AGO2 in 363 gastric cancer samples uncovered a significant correlation between tumor cell differentiation and lymph node invasion (Figure 3d) [101]. Despite the described upregulation of AGO2 in gastric cancer, decreased expression of AGO2 was found in HER-2-positive cases [101], adding complexity to the potential application of AGO2 for grading gastric cancer patients. Moreover, augmented expression of AGO2 correlated with advert prognosis of glioma cases [102]. The assessment of AGO2 mRNA and protein levels in 129 glioma specimens uncovered the correlation between elevated expression of AGO2 and lower overall survival, as well as progression-free survival (Figure 3f) [102]. In addition, it was shown that AGO2 level increased during glioma progression [102].

The prognostic value of AGO1 has been implicated in colon cancer [103]. The upregulation of AGO1 was inversely correlated with survival rates of colon cancer patients, based on the assessment of 75 cases (Figure 3e) [103]. AGO1 was also suggested as the biomarker of head and neck squamous cell carcinoma, when analysis of 21 tumor tissues revealed a significant upregulation of the *AGO1* gene expression (Figure 3g) [104]. Interestingly, for 3 of the assessed 21 cases, amplification of *AGO1* was observed [104].

Despite the large body of data documenting upregulation of the AGOs in numerous types of cancer, melanoma tumors are characterized by lower expression of AGO2 compared to other neoplastic transformations, or even normal tissues (Figure 3h) [7]. Even more striking, for other skin cancers, i.e., actinic keratoses, basal cell carcinomas, and squamous cell carcinomas, the overexpression of AGO1 and AGO2 was instead observed [105]. The decrease in AGO2 in cancer cells vs. normal controls was also found for childhood acute lymphoblastic leukaemia, which was based on the analysis of 25 cases [106]. Additionally, the decline in AGO2 levels were associated with progression of the disease (Figure 3i) [106]. A similar correlation between AGO2 expression and tumorigenesis has been implicated for clear cell renal cell carcinoma [107]. The evaluation of mRNA expression data from The Cancer Genome Atlas revealed decreased levels of AGO2 in clear cell renal cell carcinoma tumors as compared to normal tissues (Figure 3j) [107].

The relationship of AGO3 and AGO4 expression with tumorigenesis has not been frequently reported. However, the decreased expression of *AGO3* and *AGO4* genes was reported for primary hepatocellular carcinomas as compared to healthy tissues (Figure 3k) [108]. On the contrary, colon cancer tissues augmented AGO3 and AGO4, along with AGO2, and higher levels were found as compared to normal controls (Figure 3e) [103]. Furthermore, elevated *AGO2–4* expression correlated with distant metastases of colon tumors [103].

### 4.2. The Biomarker Potential of Modifications of AGO Proteins

The level of activity and specific functions of AGO proteins are controlled not only via regulation at the protein level, but also by various PTMs [48]. The modifications of AGO proteins significantly affect the involvement of proteins in tumor associated processes; therefore, the possible biomarker potential of AGO PTMs in neoplasia has been documented. For instance, high levels of acetylated AGO2 was correlated with poor prognosis for lung cancer patients [53]. Another AGO2 PTM, phosphorylation at Y393, was also found to be an adverse prognosis factor, in this case for breast cancer [48]. The association between the other AGO proteins and PTMs in cancer progression and prognosis, however, remains largely elusive.

### 4.3. The Biomarker Potential of Genetic Variations of AGO Proteins

Several single nucleotide polymorphisms (SNPs) of *AGO1* and *AGO2* genes have been described in a substantial number of neoplastic vs. control case studies (Table 1). For instance, genotyping of 855 nasopharyngeal carcinoma samples revealed 25 *AGO2* SNPs, of which one (rs3928672 GA + AA), was associated with significantly increased risk of the disease [109]. Moreover, the polymorphism was also correlated with elevated AGO2 expression levels in cancer tissues [109]. The impact of *AGO2* SNPs on disease susceptibility has also been assessed in breast cancer by different research groups. Based on the analysis of 488 breast cancer specimens, Sung and colleagues depicted that two *AGO2* genetic variants, rs11786030 A/G and rs2292779 C/G, were correlated with elevated risk of breast cancer [110]. In like manner, the polymorphism of the AGOs encoding genes in breast cancer cases was analyzed for 417 Russian patients [111]. The study did not signal a significant impact of *AGO2* SNPs; however, rs595055 C/T, one of the *AGO1* genetic variants, was associated with augmented breast cancer risk [111]. Further studies on the influence of *AGO1* and *AGO2* SNPs on breast cancer susceptibility were conducted on 93 Mediterranean cases and uncovered another *AGO1* SNP (rs636832 A/A), as well as one more *AGO2* (rs2977490 G/G) variant associated with elevated risk of the disease [112].

A series of case vs. control studies implicated the possible application of *AGO1* genotyping in cancer risk assessment. Analysis of *AGO1* genetic variations of 628 Chinese gastric cancer specimens uncovered lower disease susceptibility for individuals bearing the rs636832 AA + A variant [113]. *AGO1* polymorphisms have also been assessed as an influence on lung cancer risk. Based on the analysis of 473 Chinese patients’ DNA, the rs595961 AG genetic variant of *AGO1* was found to correlate with elevated susceptibility of lung carcinomas [114]. Further genotyping experiments, including 622 Korean lung cancer cases, revealed a protective effect of the rs636832 A  >  G *AGO1* variant [115]. Another neoplasia, partially facilitated by specific genetic variants of *AGO1,* is clear cell renal cell carcinoma. *AGO1* rs595961 AG + GG genotype was found to decrease the odds ratio of the disease for 279 American male subjects [116].

There is a significant amount of literature concerning the impact of polymorphisms on AGO encoding genes and the risk of cancer; therefore, Dobrijevic et al. performed a meta-analysis to seek a general influence of *AGO1* and *AGO2* SNPs on neoplasia susceptibility based on eleven reports [98,109,113,114,115,116,117,118,119,120,121]. Two genetic variants of *AGO1* (rs636832, rs595961) and a single *AGO2* SNP (rs4961280) were analyzed [122]. The most striking results were achieved for the *AGO1* rs636832 GA genetic variant, which was found to hold a protective effect on overall cancer risk [122]. Moreover, another of the analyzed *AGO1* SNPs variant, rs595961 AG, was implicated as a cancer risk factor for the Asian population [122].

## 5. AGO Proteins as Potential Therapeutic Applications

The engagement of RNAi in therapeutic strategy is an exciting and promising perspective. To date, two RNAi-based drugs, ONPATTRO and GIVLAARI, have been approved by the FDA for the treatment of acute hepatic porphyria and peripheral nerve disease, respectively [123]. Cancer development is driven by overexpression or aberrant activation of proto-oncogenes; therefore, various therapeutics taking advantage of endogenous RNAi machinery, specifically targeting transcripts upregulated in cancer cells, have been developed and are currently undergoing clinical trials [124]. Given the efforts put into delineation of the detailed mechanisms of RNAi, development of novel RNAi-based approaches and methods of delivery, we are on the verge of broad engagement of RNAi for anti-cancer therapies.

Besides the potential of RNAi phenomena per se for the development of novel therapeutic strategies, some reports also point to the feasibility of manipulating AGO activity for reducing tumor growth. In particular, the small molecule TPE, which inhibits the association between AGO2 and miRNAs, was shown to suppress growth of 3T3 mouse embryonic cells and completely inhibit tumor formation in mouse models in vivo [125]. The potential of this therapeutic approach was further indicated when targeting the binding domain of AGO proteins with a small molecular inhibitor, which resulted in enhanced granulocytic differentiation of promyelocytic leukaemia cell line NB4 upon treatment with retinoid acid [126].

miRNA profiling from serum is a promising tool for routine check-up of patient response to therapy, especially given the simplicity of collecting the input patient material. Fuji and colleagues described a novel option for monitoring the outcome of colorectal cancer patients based on the levels of circulating AGO2:miR-21/200c complexes [127]. Therefore, AGO proteins can also be utilized to enhance the biomarker potential of circulating miRNAs.

Although numerous miRNA and siRNA molecules exhibit potential to reduce tumor growth, efficient and safe modes of delivery remain a limiting step for the broad application of RNAi in clinics. However, usage of AGO2-conjugated nanoparticles to deliver the tumor suppressor miR-376 into the xenograft mouse tumor was shown to be an efficient and non-toxic strategy [128], demonstrating that miRNAs/siRNAs could be targeted for cancer therapies through the AGOs.

## 6. Conclusions

The tight regulation of RNAi activity is necessary in order to ensure normal development of the human body. Even minute dysregulations of miRNA molecules and proteins engaged in RNAi may constitute the basis of severe malignancies, including cancer. Although small RNA functions and their potential application in neoplasia has been greatly explored in the last 20 years, the studies on their partner proteins, AGO1–4, and the involvement in cancer development has largely lagged behind. However, due to the publication of numerous reports on AGO1–4 function in tumorigenesis, new light has been shed on the mediators of RNAi processes in the context of malignant tissues. Given the impact of the presented advances in the delineation of AGO1 and AGO2 dysregulation and activity in cancers of various origin, the broad function of the AGOs in neoplastic transformation will be further documented in the foreseeable future. Understanding of AGO1–4 involvement in tumorigenesis will most probably be followed by the clinical application of the knowledge to aid the development of RNAi-based anti-cancer strategies.

## Figures and Tables

**Table 1:** The impact of AGO polymorphisms on cancer susceptibility.

AGO	SNP	Cancer Type	Odd Ratio	Reference
AGO1	rs595055 C/T	Breast cancer	Increased	[111]
	rs636832 A/A	Breast cancer	Increased	[112]
	rs636832 AA + A	Gastric cancer	Decreased	[113]
	rs636832 A > G	Lung cancer	Decreased	[115]
	rs595961 AG	Lung cancer	Increased	[114]
	rs595961 AG + GG	Clear cell renal cell carcinoma	Decreased	[116]
	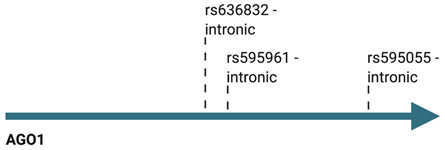			
AGO2	rs3928672 GA + AA	Nasopharyngeal carcinoma	Increased	[109]
	rs11786030 A/G	Breast cancer	Increased	[110]
	rs2292779 C/G	Breast cancer	Increased	[110]
	rs2977490 G/G	Breast cancer	Increased	[112]